# An Oleuropein β-Glucosidase from Olive Fruit Is Involved in Determining the Phenolic Composition of Virgin Olive Oil

**DOI:** 10.3389/fpls.2017.01902

**Published:** 2017-11-07

**Authors:** David Velázquez-Palmero, Carmen Romero-Segura, Rosa García-Rodríguez, María L. Hernández, Fabián E. Vaistij, Ian A. Graham, Ana G. Pérez, José M. Martínez-Rivas

**Affiliations:** ^1^Department of Biochemistry and Molecular Biology of Plant Products, Instituto de la Grasa (CSIC), Sevilla, Spain; ^2^Centre for Novel Agricultural Products, Department of Biology, University of York, York, United Kingdom

**Keywords:** β-Glucosidase, *Olea europaea*, oleuropein, olive fruit, phenolic compounds, virgin olive oil

## Abstract

Phenolic composition of virgin olive oil is determined by the enzymatic and/or chemical reactions that take place during olive fruit processing. Of these enzymes, β-glucosidase activity plays a relevant role in the transformation of the phenolic glycosides present in the olive fruit, generating different secoiridoid derivatives. The main goal of the present study was to characterize olive fruit β-glucosidase genes and enzymes responsible for the phenolic composition of virgin olive oil. To achieve that, we have isolated an olive β-glucosidase gene from cultivar Picual (*OepGLU*), expressed in *Nicotiana benthamiana* leaves and purified its corresponding recombinant enzyme. Western blot analysis showed that recombinant OepGLU protein is detected by an antibody raised against the purified native olive mesocarp β-glucosidase enzyme, and exhibits a deduced molecular mass of 65.0 kDa. The recombinant OepGLU enzyme showed activity on the major olive phenolic glycosides, with the highest levels with respect to oleuropein, followed by ligstroside and demethyloleuropein. In addition, expression analysis showed that olive *GLU* transcript level in olive fruit is spatially and temporally regulated in a cultivar-dependent manner. Furthermore, temperature, light and water regime regulate olive *GLU* gene expression in olive fruit mesocarp. All these data are consistent with the involvement of OepGLU enzyme in the formation of the major phenolic compounds present in virgin olive oil.

## Introduction

Olive *(Olea europaea* L.) is one of the first plants grown as oil crop. Consequently, olive oil is one of the oldest known plant oils and it can be consumed as virgin olive oil (VOO). In the Mediterranean diet, this oil constitutes the main lipid source and it has been related with several beneficial nutritional properties which are mainly associated to its phenolic components (Konstantinidou et al., 2010; Visioli and Bernardini, 2011). However, phenolic compounds are relevant not only because their nutritional properties, but also due to their organoleptic characteristics. In fact, phenolic components are involved in the pungent and bitter sensory notes of VOO (Andrewes et al., 2003; Mateos et al., 2004). Phenolic compounds are being currently used as a trait in new cross breeding programs (León et al., 2011), and also as VOO quality markers, because of their health promoting and organoleptic properties.

Oleuropein, demethyloleuropein and ligstroside, the most significant phenolic glycosides detected in the olive fruit, belong to the secoiridoids class, a group of monoterpenoids typical of the Oleaceae family with a cleaved methylcyclopentane skeleton (Obied et al., 2008). On the contrary, the main phenolic compounds detected in VOO are the secoiridoid derivatives, resulting from the enzymatic hydrolysis of these olive fruit glycosides. Specifically, the aldehydic forms of oleuropein and ligstroside aglycones (3,4-DHPEA-EA and *p-*HPEA-EA, respectively), and the dialdehydic forms of decarboxymethyloleuropein and ligstroside aglycones (3,4-DHPEA-EDA and *p-*HPEA-EDA, respectively) (Montedoro et al., 2002). Oleuropein derivatives exhibit the highest antioxidant activity (Ramos-Escudero et al., 2015), protein-denaturing/protein-cross-linking properties (Konno et al., 1999), cytotoxic effects (Bernini et al., 2011) and effectivity as chronic disease preventive agents (Pinto et al., 2011).

The phenolic profile of VOO is mainly derived from the amount of phenolic glycosides originally found in the tissues of olive fruit and the activity of oxidative and hydrolytic enzymes operating on these glycosides during VOO processing (García-Rodríguez et al., 2011; Romero-Segura et al., 2012). Although secoiridoids biosynthesis and degradation pathways are still not fully understood (Obied et al., 2008), hydrolysis by highly specific β-glucosidases seems to be critical for the diverse roles attributed to secoiridoid derivatives. In this sense, the wide array of physiological roles assigned to plants β-glucosidases (β-d-glucoside glucohydrolases, EC 3.2.1.21), such as functions in plant secondary metabolism, symbiosis, defense, signaling, and cell wall lignification and catabolism, are determined by their tissue and subcellular localization, and their substrate-specificities (Cairns and Esen, 2010).

The existence of various β-glucosidase isoforms in olive was first reported by Mazzuca et al. (2006), who described the localization of two isoforms of oleuropein-degradative β-glucosidases in the oil droplets and in the chloroplasts of mesocarp of green olive fruits. Transcriptomic (Alagna et al., 2012) and proteomic (Bianco et al., 2013) studies confirm that olive, similar to most plants, possesses several distinct β-glucosidases. Recently, the isolation and characterization of a defense-related β-glucosidase gene from olive (cv. Koroneiki) has been described (Koudounas et al., 2015). Nearly all these earlier reports on this enzyme have been centered in its physiological function as a defense mechanism which specifically generates oleuropein-derived compounds with established antimicrobial activities. In contrast, no similar studies have been carried out on the β-glucosidase genes/enzymes in relation to the VOO quality; despite that this knowledge may be very valuable to enhance marker assisted breeding programs to obtain new varieties with tailored oil quality characteristics.

We have previously isolated and purified to apparent homogeneity a protein with β-glucosidase activity from olive fruit mesocarp which exhibits high activity with the main phenolic glycoside in olive fruit, oleuropein, and gives rise to one of the most important phenolic compounds in VOO (3,4-DHPEA-EA) as the main reaction product (Romero-Segura et al., 2009). Data on the β-glucosidase activity during ripening of olive fruit from cultivars Arbequina and Picual are in good agreement with the phenolic composition of the oils obtained from fruits with different degrees of maturity (Romero-Segura et al., 2012).

The objective of this study was to characterize olive fruit β-glucosidase genes and enzymes responsible for the phenolic composition of VOO. Thus, we have isolated an olive β-glucosidase gene from cultivar Picual, which codes for an enzyme that displays the highest activity toward oleuropein. The immunological and catalytic properties of this olive β-glucosidase enzyme, together with its expression data, are in agreement with its participation in the biosynthesis of the major phenolic compounds found in VOO.

## Materials and Methods

### Plant Material

Olive (*Olea europaea* L. cv. Picual and Arbequina) trees were cultivated in the experimental orchard of Instituto de la Grasa, Sevilla (Spain), with drip irrigation and fertirrigation from the time of flowering to fruit ripening. In the case of non-irrigated treatment, the olive trees received only natural rainfall.

Young drupes, developing seeds, and mesocarp tissue were harvested at different weeks after flowering (WAF) corresponding to different developmental stages of the olive fruit: green (9, 12, 16, and 19 WAF); yellow-green (23 WAF); turning or veraison (28 and 31 WAF); and mature or fully ripe (35 WAF). Immediately after harvesting, olive tissues were frozen in liquid nitrogen, and stored at -80°C.

Stress treatments were carried out according to Hernández et al. (2011). Olive branches with approximately 100 olive fruit at turning stage (28 WAF) were taken from olive trees and incubated in a growth chamber at 25°C with a 12 h light/12 h dark cycle to imitate physiological conditions of the tree. The light intensity was 11.5 μmol m^-2^ s^-1^. For stress experiments, standard conditions were modified according to the effect studied. For low and high temperature treatments, the branches with olive fruit were incubated at the standard light intensity, at 15 or 35°C, respectively. To evaluate the effect of the darkness, the standard temperature was maintained, and light was turned off. To study the effect of wounding, the whole surface of the olive fruit was mechanically damaged with pressure at zero time using forceps with serrated tips, affecting mesocarp tissue. To maintain the natural photoperiod day/night of the olive fruit, the zero time of each experiment was selected 2 h after the start of the light period. When indicated, olive mesocarp tissues were collected, frozen in liquid nitrogen, and kept at -80°C.

### Isolation of a β-Glucosidase Full-Length cDNA Clone

Candidate sequences for olive β-glucosidases were identified in the olive ESTs database (Muñoz-Mérida et al., 2013) by means of the tblastn algorithm together with amino acid sequences of known plant β-glucosidase proteins. One of them, which showed high expression levels in mesocarp tissue according to *in silico* expression analysis, was selected for cloning. Based on this sequence, a specific pair of primers CR3 5′-AAGAGCACCAAAGTCTGCAATG-3′ and CR4 5′-GGAGCCCAACTCCTTTATTGG-3′ was designed. These primers, together with an aliquot of an olive Uni-ZAP XR cDNA library constructed with mRNA isolated from 13 WAF olive fruit of cultivar Picual (Haralampidis et al., 1998), were used for PCR amplification. The generated DNA fragment was subcloned into the vector pSpark^®^ I (Canvax, Spain) and sequenced in both directions. DNA sequence determination and analysis was carried out as described in Hernández et al. (2016).

### Total RNA Extraction and cDNA Synthesis

1–2 g of frozen olive tissues harvested from at least three different olive trees, were used for total RNA isolation as described by Hernández et al. (2005). Verification of RNA quality, removal of genomic DNA and cDNA synthesis were performed according to Hernández et al. (2009).

### Quantitative Real-Time PCR (qRT-PCR)

Gene expression analysis was performed by qRT-PCR using an Mx3000P^TM^ real-time PCR System and the “Brilliant^®^ SYBR^®^ Green Q-PCR Master Mix (Stratagene, La Jolla, CA, United States) as previously described (Hernández et al., 2009). Primer3 program^1^ was used to design primers for gene-specific amplification (Supplementary Table S1). The housekeeping olive ubiquitin2 gene (*OeUBQ2*, AF429430) was used as an endogenous reference to normalize. The real-time PCR data were calibrated relative to the corresponding gene expression level in 12 WAF mesocarp tissue from Picual in the case of tissue and developmental expression studies, whereas for the stress studies the data were calibrated relative to the corresponding gene expression level at zero time for each treatment and cultivar. In both cases, the 2^-Δ Δ*C*_T_^ method for relative quantification was followed (Livak and Schmittgen, 2001). The data are presented as means ± standard deviation (SD) of three different qRT-PCR reactions carried out in three different 96-well plates. Each reaction was performed in duplicate in each plate.

### Transient Expression of *OepGLU* Gene in *Nicotiana benthamiana*

For functional *Agrobacterium*-mediated CaMV35S-driven transient expression, the *OepGLU* coding sequence was PCR-amplified using the specific primers YDV1F (5′-CACCATGGATATCCAAAGCAAC-3′) and YDV1R+His (5′-CTAGTGATGGTGATGGTGATGCCCGGTGCTGCCTCTAAGCCTTTTAC-3′), and subcloned into the GATEWAY^®^-compatible binary vector pH2GW7 (Karimi et al., 2002). The resulting purified pH2GW7-OepGLU construct was used to transform *Agrobacterium tumefaciens* strain GV3101 using freeze-thaw method described by Höfgen and Willmitzer (1988). *Nicotiana benthamiana* leaves were pressure infiltrated with *A. tumefaciens* cultures (OD_600_ approximately 1.0) as described by Popescu et al. (2007). Samples were collected 3 days after infiltration, frozen in liquid nitrogen and kept at -80°C.

### Purification of OepGLU Recombinant Isoenzyme Expressed in *N. benthamiana* Leaves

To obtain the crude extract, 4 g of infiltrated leaf tissue were thawed and homogenized in 30 ml of 20 mM Na-phosphate buffer pH 7.4 containing 500 mM NaCl, 20 mM imidazole, 5% (w/v) polyvinyl polypyrrolidone, and 1 mM phenylmethanesulfonyl fluoride using an Ultraturrax homogenizer at 4°C. The resulting homogenate was centrifuged at 27000 *g* for 20 min at 4°C. The clear supernatant was filtered through three layers of Miracloth (Calbiochem, United States) and was used as the crude extract.

To purify the recombinant OepGLU protein containing the C-terminal 6xHis tag motif, 30 ml of crude extract was loaded onto a 1-ml His GraviTrap column (GE Healthcare, United Kingdom), and OepGLU protein was eluted with 3 ml of 20 mM Na-phosphate pH 7.4 containing 500 mM NaCl and 500 mM imidazole. Remaining NaCl and imidazole were removed by means of a PD-10 column (GE Healthcare, United Kingdom). The enzymatic solution was concentrated in 30 kDa microcentrifuge filters Vivaspin^®^ (GE Healthcare, United Kingdom) at 2000 *g* and 4°C to a final volume of 250 μl. This purified and concentrated preparation was used for OepGLU biochemical characterization.

### β-Glucosidase Assay

Two methods for *in vitro* assaying β-glucosidase activity were used in this study (Romero-Segura et al., 2009). A spectrophotometric method, in which the β-glucosidase activity was determined by continuously monitoring the increase in absorbance at 405 nm related to the increasing amount of *p*-nitrophenol liberated from the synthetic glucoside pNPG, and a second method based on the direct determination of the hydrolyzed natural olive glucoside, oleuropein, by HPLC analysis.

### HPLC Analysis

Analytical HPLC of phenolic compounds was performed in a Beckman Coulter liquid chromatographic system equipped with a System Gold 168 detector, a solvent module 126 and a Mediterranea Sea 18 column (4.0 mm i.d. x 250 mm, particle size = 5 μm) (Teknokroma, Spain). Quantification and identification of phenolic compounds was performed following a previously described methodology (Luaces et al., 2007).

### Protein Determination and Electrophoresis

The protein concentration was estimated using the Bio-Rad (United States) Bradford protein reagent dye with BSA as standard. SDS-PAGE was performed as previously described (Romero-Segura et al., 2009).

### Preparation of Anti-β-Glucosidase Polyclonal Antibodies and Immunoblot Analysis

Polyclonal antibodies against the native olive β-glucosidase protein purified from olive mesocarp according to the method described by Romero-Segura et al. (2009) were prepared in rabbit by Production and Animal Experimentation General Service of the University of Seville.

For Western blotting, 5–6 μg of protein samples were separated by SDS-PAGE as described above and electro-blotted onto nitrocellulose membrane using the Mini Trans-Blot^®^ system (Bio-Rad). Bound anti-β-glucosidase primary antibody was detected using an anti-rabbit alkaline phosphatase-conjugated secondary antibody (Sigma–Aldrich, United States). When a mouse monoclonal anti-6xHis antibody (GE Healthcare) was used as primary antibody, an anti-mouse alkaline phosphatase-conjugated antibody (Invitrogen) was employed as secondary antibody. To detect alkaline phosphatase activity after antibodies incubation, nitrocellulose membrane was submerged in a solution obtained by dissolving a SIGMAFAST^TM^ BCIP^®^/NBT tablet (Sigma–Aldrich) in 10 ml of distilled water.

## Results

A number of contigs with a high degree of similarity to plant β-glucosidases were selected from the olive EST database (Muñoz-Mérida et al., 2013). Among them, one which showed high expression levels in mesocarp tissue according to *in silico* expression analysis was chosen for cloning. Two specific primers were designed on the basis of this contig sequence, and used for PCR amplification together with an aliquot of an olive cDNA library of cultivar Picual. We obtained a full-length cDNA clone of 1848 bp, which was designated *OepGLU*, and contained an open reading frame encoding a predicted protein of 551 amino acids (Supplementary Figure S1), with a calculated molecular mass of 62.8 kDa and a p*I* of 6.6. The deduced OepGLU amino acid sequence from cultivar Picual displayed a 98% identity to an olive β-glucosidase cDNA clone (AY083162) from cultivar Koroneiki (Koudounas et al., 2015).

Alignment of the deduced amino acid sequence of OepGLU from cultivar Picual with other plant β-glucosidase protein sequences (Supplementary Figure S1) suggests that it codes for a β-glucosidase enzyme because it showed the conserved sequence motifs characteristic of the glycosyl hydrolases family 1 (GH1) T(F/L/M)NEP and Y(I/V)TENG, which include the two glutamic acid residues involved in the catalytic mechanism (Esen, 2003). In addition, the conserved amino acids Gln, His, Asn, Glu and two Trp which have been shown to be essential for the binding of the glucose (Cairns and Esen, 2010) were also found. A putative *N*-glycosylation site (N83) has also been detected in the OepGLU sequence, and the presence of a conserved GH1 family domain has been identified by NCBI Conserved Domain Search and the Pfam software. Analysis of OepGLU deduced protein sequence with target prediction software such as WolfPSORT and TargetP did not give rise to a clear subcellular localization. In fact, a putative nuclear localization signal (RRKR) could be found at amino acids 543–546 and a 25 amino acid N-terminal signal peptide was also predicted (Supplementary Figure S1), generating after its proteolytic cleavage a mature protein of 526 amino acids, with a calculated molecular mass of 60.3 kDa and a p*I* of 7.0.

### Purification and Immunological Characterization of OepGLU Recombinant Enzyme

**FIGURE 1:**
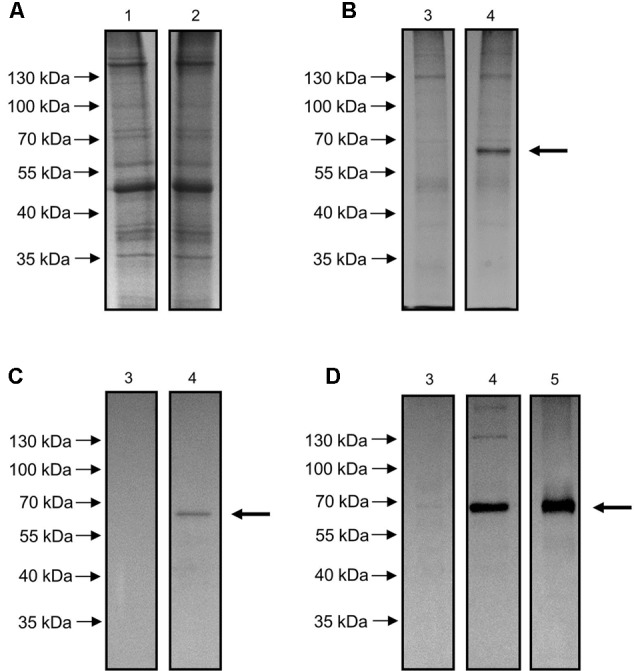
SDS-PAGE of crude extract **(A)** and purified preparation **(B)** of *N. benthamiana* leaves transiently expressing the *OepGLU* gene and Western blot analysis of purified preparation using anti-6xHis antibody **(C)** or anti-GLU antibody **(D)**. Lane 1, crude extract of infiltrated leaves with untransformed *A. tumefaciens* suspensions (control); lane 2, crude extract of infiltrated leaves with pH2GW7-OepGLU transformed *A. tumefaciens* suspensions; lane 3, purified preparation of infiltrated leaves with untransformed *A. tumefaciens* suspensions (control); lane 4, recombinant OepGLU purified preparation; lane 5, purified GLU protein according to the method of Romero-Segura et al. (2009). 6 μg of protein were loaded per lane in all cases. The primary antibody dilutions used were 1:1500 **(C)** and 1:5000 **(D)** and the secondary antibody dilution were 1:2500 **(C)** and 1:10000 **(D)**. The band corresponding to the olive β-glucosidase protein is denoted by an arrow.

To verify the functional identity of the *OepGLU* gene, *Agrobacterium*-mediated transient expression in *N. benthamiana* leaves was carried out. To that end, the *OepGLU* coding region, including a C-terminal 6xHis tag, was subcloned into the vector pH2GW7 using the GATEWAY^TM^ technology. The resultant plasmid designated pH2GW7-OepGLU+His was used to transform *A. tumefaciens* GV3101, and tobacco leaves were infiltrated with bacterial cells carrying this plasmid. Expression of the recombinant protein was optimal at 3 days after infiltration. SDS-PAGE analysis of the crude extract did not show the protein band with the expected molecular mass for the recombinant OepGLU (**Figure 1A**). However, when the enzyme preparation purified by affinity chromatography was used, an intense band with a molecular mass of 65.5 kDa was detected (**Figure 1B**, lane 4). In contrast, this protein band was not observed in purified preparations isolated from tobacco leaves infiltrated with untransformed *Agrobacterium* cells (**Figure 1B**, lane 3).

Purified preparations of recombinant OepGLU were also analyzed by western blot using the anti-6xHis antibody (**Figure 1C**) and the antibody raised against the native olive β-glucosidase (**Figure 1D**). In both cases, a protein band with the same molecular mass as deduced from the SDS-gel was observed.

### Kinetic Properties of Recombinant OepGLU in Comparison to the Native Enzyme

**FIGURE 2:**
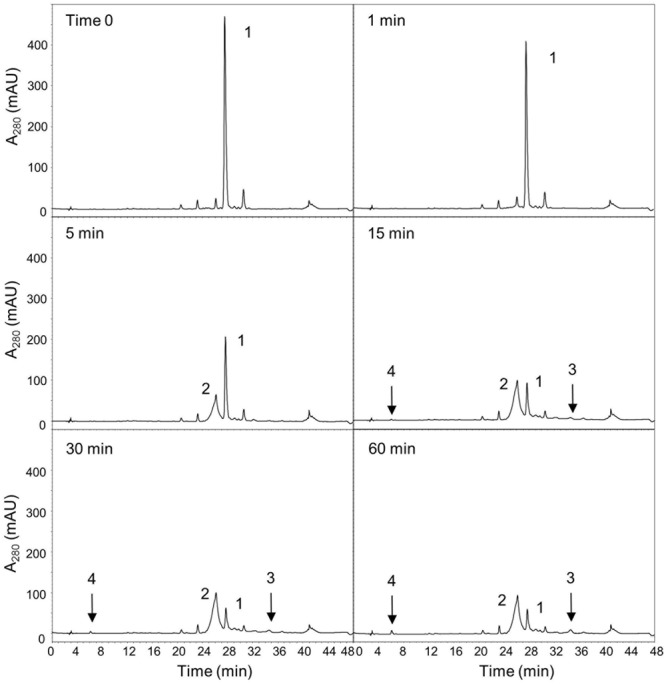
Time-course of the oleuropein hydrolysis catalyzed by the purified recombinant OepGLU. Enzyme activity was assayed as indicated in Material and Methods. Identification of peaks: 1, oleuropein; 2, OA-isomers; 3, 3,4-DHPEA-EA; 4, hydroxytyrosol.

In the present study, purified preparations of recombinant OepGLU, but not crude extracts, were able to hydrolyze the artificial substrate *p*-nitrophenyl-β-D-glucopyranoside (pNPG) and the major natural olive phenolic glycoside oleuropein in a time-dependent manner. The enzymatic hydrolysis of oleuropein by purified recombinant OepGLU was monitored for up to 60 min by HPLC analysis (**Figure 2**). More than 50% of the oleuropein initially present was hydrolyzed after 5 min, producing as the first reaction product a mixture of oleuropein aglycone isomers (OA-isomers). After 15 min, the broad peak of OA-isomers was reduced, whereas 3,4-DHPEA-EA and hydroxytyrosol began to accumulate. The purified recombinant olive β-glucosidase exhibited a specific activity of 67.6 U/mg using oleuropein as substrate.

Once it was established the capacity of the purified recombinant olive β-glucosidase to hydrolyze oleuropein, its activity was also measured using the synthetic glucoside pNPG as substrate, exhibiting a much lower specific activity (1.4 U/mg). Hence, the natural substrate oleuropein was used to perform the biochemical characterization of the recombinant olive β-glucosidase.

**FIGURE 3:**
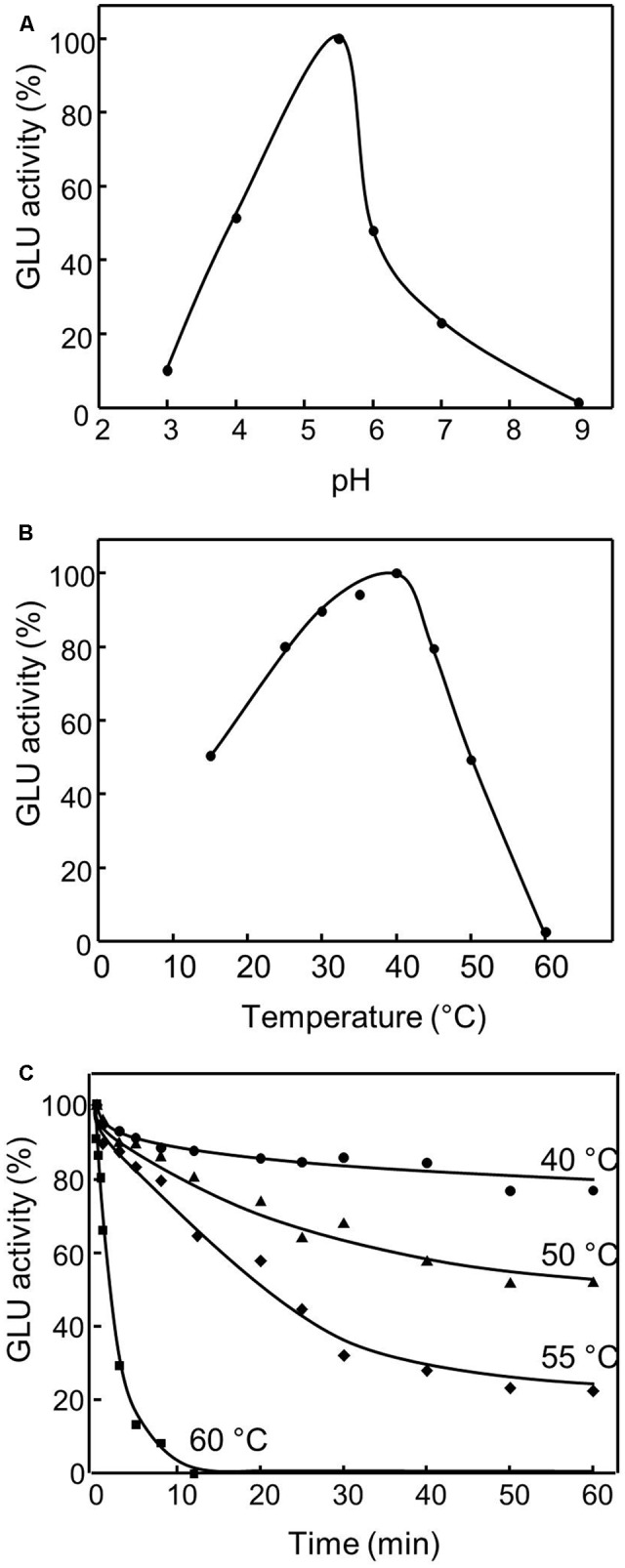
Effect of pH **(A)**, temperature **(B)**, and thermal stability **(C)** on the purified recombinant OepGLU activity. The optimum pH was determined using sodium acetate, phosphate and borate buffers (50 mM) in the standard assay. The optimum temperature was determined using a temperature interval of 15–60°C in the standard assay. Thermal stability was determined under standard assay conditions after incubation of purified preparation at different temperatures for 60 min. 100% activity was 50.9 U/ml.

The purified recombinant olive β-glucosidase displayed an optimum pH of 5.5 with a fast decrease of activity above it (**Figure 3A**), and showed > 80% of its highest activity at 25–45°C, with an optimum temperature when assayed at 40°C and a strong decline over 45°C (**Figure 3B**). Thermal inactivation kinetics showed that recombinant OepGLU was active up to 40°C with a significant decline over this temperature (**Figure 3C**).

In addition, β-glucosidase activity was assayed with a mixture of oleuropein, demethyloleuropein and ligstroside, the three most important phenolic glycosides identified in olive fruit, in order to mimic what happens during the milling step of the industrial VOO extraction process, when the enzyme and possible substrates meet as olive fruit tissues are disrupted. Substrate selectivity experiments (**Table 1**) showed that the highest activity level was reached using oleuropein as substrate, followed by ligstroside (25.6%) and demethyloleuropein (15.6%). In particular, after a 5 min reaction time it could be observed in the corresponding chromatogram that most of the oleuropein was hydrolyzed followed by ligstroside, with the consequent appearance of OA-isomers and ligstroside aglycone isomers (LA-isomers), respectively (Supplementary Figure S2). Verbascoside was used as negative control, since its chemical structure is significantly different from that of the other three phenolic glucosides and does not contain a non-reducing terminal β–D-glucosyl residue.

**Table 1:** Substrate selectivity of purified recombinant OepGLU on a mixture of various natural olive glycosides.

Substrate	Relative activity (%)
Oleuropein	100.0
Ligstroside	25.6
Demethyloleuropein	15.6

*Activity was determined by measuring the corresponding hydrolyzed natural olive glycoside by HPLC. Initial concentration of the substrates in the assay mixture was 5 mM each.*

To obtain the kinetic parameters of recombinant olive β-glucosidase, enzyme activity was measured over a range of concentrations of oleuropein as substrate (Supplementary Figure S3). The calculated *K_m_* for oleuropein was 26.8 mM and the *V_max_* 263.2 U/mg, with a catalytic efficiency (*V_max_*/*K_m_*) of 9.8.

### Tissue Specificity and Developmental Expression of Olive *GLU* Gene Is Cultivar-Dependent

**FIGURE 4:**
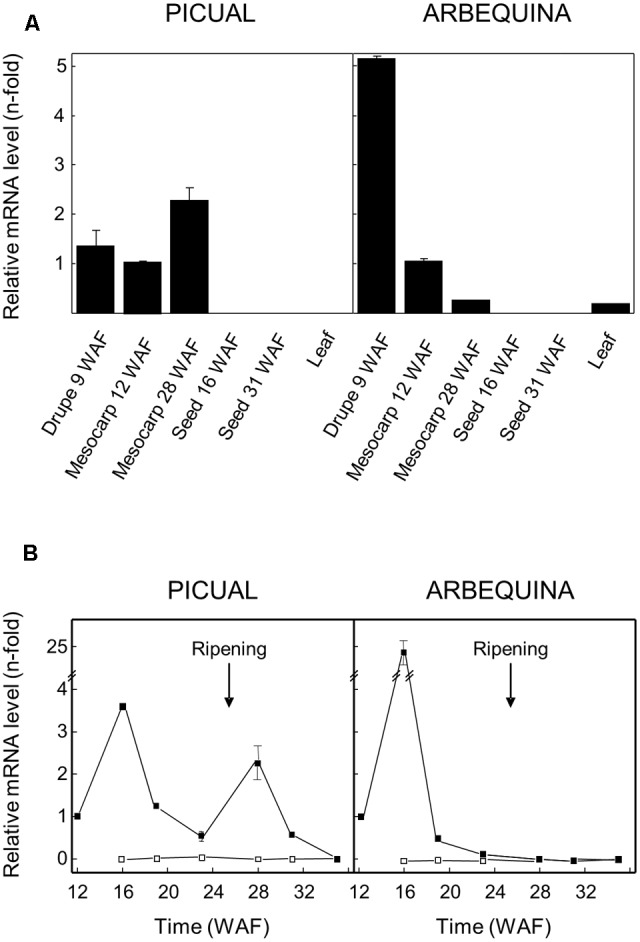
Relative expression levels of olive *GLU* gene in different tissues of Picual and Arbequina cultivars **(A)**, and in mesocarp tissue (closed squares) or seeds (open squares) during the development and ripening of olive fruit **(B)**. The beginning of fruit ripening, which coincides with the appearance of purple color, is indicated by an arrow.

Olive *GLU* gene expression levels were analyzed in different Picual and Arbequina olive tissues by qRT-PCR using specific primers (**Figure 4A**), with the aim of investigating its physiological role and its possible contribution to the content of the different phenolic compounds present in the VOO. In both cultivars, higher expression levels were detected in young drupes and mesocarp compared to seeds, where transcript levels were negligible. Young drupes of 9 WAF from cultivar Arbequina showed the highest expression level. Interestingly, transcript levels in mesocarp at turning stage (28 WAF) were much higher in Picual than in Arbequina cultivar.

Besides, olive *GLU* transcript levels were analyzed at different times during olive fruit development and ripening in Picual and Arbequina mesocarp and seed tissues (**Figure 4B**). A maximum transcription level was observed in green mesocarp (16 WAF) from both cultivars. In the case of cultivar Arbequina, the high expression level detected at 16 WAF dramatically decreased after this maximum, reaching constant low levels during the rest of the olive fruit development and ripening periods. On the contrary, in the cultivar Picual, olive *GLU* expression level showed a second maximum, lower than the first one, once the olive fruit ripening period has started (28 WAF). Unlike mesocarp tissue, olive *GLU* gene exhibited almost undetectable transcript levels in seeds from Picual and Arbequina (**Figure 4B**), which is consistent with the very low enzyme activity levels observed in both cultivars (Romero-Segura et al., 2011).

The same study was also performed in Picudo, Hojiblanca and Manzanilla cultivars, using olive fruit mesocarp at the three main stages in which olive fruit are harvested for olive oil production: yellow-green (23 WAF), turning or veraison (31 WAF), and mature or fully ripe (35 WAF) (Supplementary Figure S4). The olive *GLU* gene expression levels in the cultivars Picudo and Hojiblanca remained low, showing no significant changes. On the contrary, the transcript levels in the cultivar Manzanilla showed an increase during fruit ripening, and then decreased at the end of the ripening period.

### Transcriptional Regulation in Olive Fruit Mesocarp of *GLU* Gene in Response to Abiotic Stresses

**FIGURE 5:**
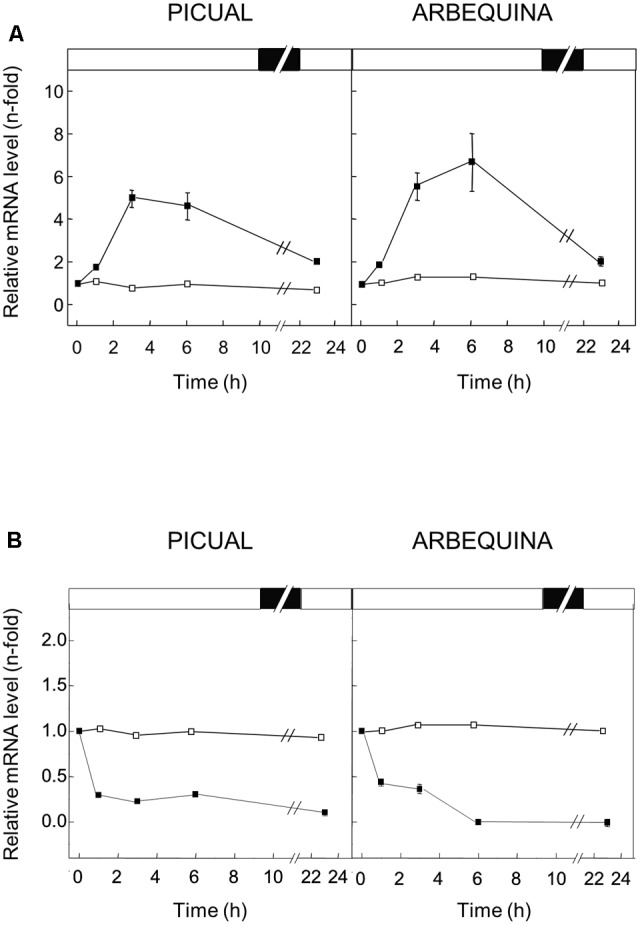
Effect of low **(A)** and high **(B)** temperature on the relative expression levels of olive *GLU* gene in the Picual and Arbequina mesocarp tissues. Branches with approximately 100 olive fruit at turning stage (28 WAF) were incubated using standard conditions (open squares), or at a temperature of 15°C **(A)** or 35°C **(B)** (closed squares). Boxes in the upper part indicate light (open) or dark (closed) periods.

**FIGURE 6:**
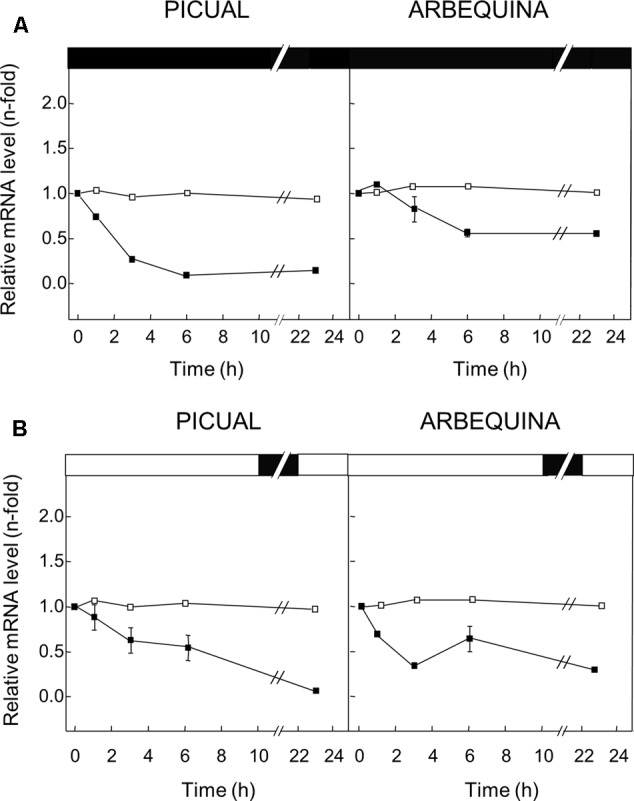
Effect of darkness **(A)** and wounding **(B)** on the relative expression levels of olive *GLU* gene in the Picual and Arbequina mesocarp tissues. Branches with approximately 100 olive fruit at turning stage (28 WAF) were incubated using standard conditions (open squares), or incubated at 25°C for 24 h in the dark **(A)** or subjected to mechanical damage and incubated at standard conditions for 24 h **(B)** (closed squares). Boxes in the upper part indicate light (open) or dark (closed) periods.

To examine the effect of different abiotic stresses on the expression level of the *GLU* gene in mesocarp tissue, olive tree branches from Picual and Arbequina cultivars with olive fruit at turning stage (28 WAF) were incubated for 24 h modifying the standard conditions (25°C with a 12 h light / 12 h dark cycle) dependent on the effect to be tested. No changes in the olive *GLU* gene expression levels were observed in olive fruit mesocarp when standard conditions were used (**Figures 5, 6**).

When low temperature (15°C) was used to incubate the olive fruit, a significant transient increase in the expression levels of olive *GLU* was observed in both cultivars, with a maximum after 3 or 6 h of treatment for Picual and Arbequina cultivars, respectively (**Figure 5A**). On the contrary, the incubation at high temperature (35°C) of olive fruit brought about a reduction in the olive *GLU* gene transcript levels in both cultivars especially after 1 h of treatment, reaching almost undetectable transcript levels after 24 h (**Figure 5B**).

To examine the effect of darkness on the transcript levels of the olive *GLU* gene in Picual and Arbequina mesocarp tissues, branches were incubated for 24 h at 25°C in the darkness. A decrease in the olive *GLU* gene expression levels was detected in both cultivars, mainly in Picual during the first 3 h of incubation (**Figure 6A**).

In addition, the potential involvement of olive *GLU* in the transcriptional response to wounding was tested in olive fruit subjected to mechanical damage from olive branches incubated at standard conditions. In this case, olive *GLU* gene expression levels declined progressively in both cultivars (**Figure 6B**).

**FIGURE 7:**
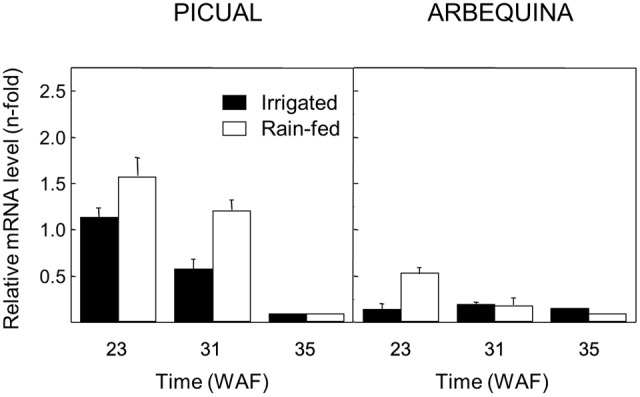
Effect of the water regime on the relative expression levels of the olive *GLU* gene in the Picual and Arbequina mesocarp tissues from olive trees cultivated with natural rainfall or irrigation.

On the other hand, since a number of studies point out that different water regimes could affect the phenolics content of VOO, showing a negative correlation between the content of secoiridoid derivatives and the water amount used for olive growing (Gómez-Rico et al., 2006; Servili et al., 2007), the effect of water regime on the transcript levels of olive *GLU* was investigated in olive fruit mesocarp of Picual and Arbequina cultivars grown with natural rainfall or irrigation. A higher transcript level was detected for the olive *GLU* gene when Picual and Arbequina were cultivated with natural rainfall only (**Figure 7**), except for late ripening stages (35 WAF) where transcript levels were very low in both watering conditions.

## Discussion

Several candidate olive β-glucosidase sequences were identified from an olive ESTs database (Muñoz-Mérida et al., 2013). This is consistent with the numerous β-glucosidase genes usually detected in the same plant, as reported in Arabidopsis (Xu et al., 2004). Since higher β-glucosidase activity levels have been observed in olive fruit mesocarp in comparison to seeds (Romero-Segura et al., 2011), one of the contigs which showed high expression levels in mesocarp tissue compared to seeds according to *in silico* expression analysis, was selected for cloning. Sequence analysis of the β-glucosidase gene isolated from olive (cv. Picual) showed that its deduced amino acid sequence contains the conserved sequence motifs and domains characteristic of the glycosyl hydrolases family 1 (GH1) (Esen, 2003; Cairns and Esen, 2010), and suggests that it codes for a β-glucosidase enzyme.

In order to characterize the immunological and kinetic properties of the olive β-glucosidase recombinant enzyme, transient expression in *N. benthamiana* leaves was performed. The band corresponding to the recombinant olive β-glucosidase was observed by SDS-PAGE only when the enzymatic preparation purified by affinity chromatography was applied, but not in the case of the purified preparation isolated from tobacco leaves infiltrated with untransformed *Agrobacterium* cells used as control, or when the crude extract was loaded onto the gel. In addition, western blot analysis of the purified preparations was performed using two types of antibodies. In the first case, an anti 6xHis antibody detected a band with identical molecular mass (65.0 kDa) than that observed by SDS-PAGE, confirming that it corresponded to the recombinant enzyme. This molecular mass of OepGLU is in the range of 55–65 kDa described for almost all plant β-glucosidase monomers (Esen, 2003), and is identical to that of 65.4 kDa reported for the β-glucosidase protein purified from olive fruit mesocarp (Romero-Segura et al., 2009). In the second case, an antibody raised against the native olive β-glucosidase protein purified from olive mesocarp (Romero-Segura et al., 2009) detected a band with identical molecular mass. Furthermore, when a preparation corresponding to native enzyme purified according to the method of Romero-Segura et al. (2009) was used as positive control, an intense band with a similar apparent size was detected. All these data strongly suggest that the recombinant OepGLU corresponds to the native β-glucosidase enzyme previously purified by our group from olive fruit mesocarp, which has been demonstrated to play a key role in shaping the VOO phenolic composition (Romero-Segura et al., 2012). Interestingly, although native and recombinant olive β-glucosidase enzymes exhibit similar molecular masses, the occurrence of post-translational modifications cannot be discarded. In fact, a unique *N*-glycosilation site was predicted in the OepGLU amino acid sequence.

Although the capacity of the recombinant olive β-glucosidase to hydrolyze oleuropein has been previously demonstrated using crude extracts from *N. benthamiana* leaves (Koudounas et al., 2015), a quantitative *in vitro* enzymatic assay using purified recombinant protein, to avoid interferences of metabolites and enzyme activities present in the tobacco leaves crude extract, has not been reported so far. Furthermore, a comprehensive characterization of the kinetic properties of the enzyme has not been carried out up to date. Purified preparations of recombinant OepGLU exhibited β-glucosidase activity with both, the artificial substrate pNPG and the major natural olive phenolic glucoside oleuropein. This result demonstrates that the *OepGLU* gene code for a β-glucosidase enzyme, confirming its functional identity. Interestingly, purified recombinant OepGLU showed very low activity levels when pNPG was used as substrate, as previously reported for the native enzyme from olive (Romero-Segura et al., 2009) and privet tree (Konno et al., 1999). It has been described for plant β-glucosidases that there is not a correspondence between the activity levels exhibited using non-physiological substrates such as pNPG, and those obtained using their natural substrates (Cairns et al., 2015). The comparison of the data obtained from the time-course of the oleuropein hydrolysis catalyzed by the purified recombinant OepGLU, with those previously reported using the purified native enzyme (Romero-Segura et al., 2012), shows that the native olive β-glucosidase hydrolyzes oleuropein more efficiently. These discrepancies on the relative activity of β-glucosidase proteins purified from the native plant and the corresponding recombinant proteins have been previously described (Himeno et al., 2013). OA-isomers are the first reaction products formed by the recombinant OepGLU after the hydrolysis of the glucoside. The elimination of the glucose molecule could destabilize the phenolic aglucone and during the reaction course the formed isomers tend to its stabilization yielding 3,4-DHPEA-EA, which simultaneously produce hydroxytyrosol by chemical hydrolytic reactions, since the recombinant β-glucosidase is the only enzyme present in the reaction mixture.

The purified recombinant OepGLU displays the highest activity at pH 5.5 and 40°C, similar values to those reported for the olive native β-glucosidase enzyme (Romero-Segura et al., 2009). Optimum pH in the range of 4.5-5.5 has been described for other β-glucosidases from plants such as rice (Akiyama et al., 1998) and *Citrus sinensis* (Cameron et al., 2001). With respect to optimum temperature, data in the interval of 40-50°C has been previously reported for the β-glucosidases from maize (Esen, 1992) and *Citrus sinensis* (Cameron et al., 2001). The recombinant olive β-glucosidase exhibits a high thermostability up to 40°C, as reported for the native enzyme from olive (Romero-Segura et al., 2009). Taking into account its thermal resistance profile, the OepGLU enzyme could act during the malaxation step of the industrial process to obtain VOO, where temperatures higher than 30°C are not unusual. However, it has been reported that after 15 min of malaxation at this temperature, no β-glucosidase activity could be detected in the paste, likely due to the presence of enzyme inhibitors (García-Rodríguez, 2014). In the same way, the thermal resistance of OepGLU could explain why the thermal treatment of olive fruit at temperatures of 56–68°C just before the milling step causes a high decrease in the content of the secoiridoid derivatives in the VOO (Yousfi et al., 2010), since at those temperatures the olive β-glucosidase enzyme should be inactivated, highly reducing the degree of hydrolysis of the phenolic glucosides. Substrate selectivity experiments showed that the recombinant olive β-glucosidase exhibits a higher preference for oleuropein as substrate, followed by ligstroside and demethyloleuropein. These results are in agreement to those reported for the native enzyme (Romero-Segura et al., 2009), and demonstrate the capacity of the recombinant OepGLU to hydrolyze the three main phenolic glucosides of olive fruit, which are the precursors of the main secoiridoid derivatives present in the VOO. The recombinant olive β-glucosidase showed kinetic parameters of *K_m_* for oleuropein (26.8 mM) and *V_max_* (263.2 U/mg), different of those described (3.8 mM and 2,500 U/mg, respectively) for the native enzyme (Romero-Segura et al., 2009), and indicate a lower catalytic efficiency of the recombinant enzyme as previously mentioned.

Plant *GLU* genes are developmentally regulated (Morant et al., 2008), and exhibit different spatial expression patterns depending on their physiological functions. In this sense, olive *GLU* gene showed different transcript levels in the studied tissues, showing its spatial regulation. Besides, changes in the *GLU* transcript level reveal that this gene is also temporally regulated, and moderately correlate with changes in the β-glucosidase activity levels in Picual and Arbequina previously reported (Romero-Segura et al., 2012). This minor discrepancy observed between transcript and activity levels could be explained by the occurrence of post-translational modifications of the olive β-glucosidase enzyme such as *N*-glycosilation, as previously mentioned. In fact, *N*-glycosilation of plant β-glucosidases has been widely described (Morant et al., 2008). Furthermore, the contribution of other olive β-glucosidases isoforms to the enzyme activity levels observed cannot be discarded. Interestingly, previous studies on these two cultivars have demonstrated significant differences not only in terms of β-glucosidase activity but also in their phenolic profiles along fruit ripening, with Picual oils being described as a VOO with medium-high phenolic content at any ripening stage while Arbequina oils typically have medium-low concentration of phenolic compounds (Romero-Segura et al., 2012). Moreover, expression data from cultivars Picudo, Hojiblanca and Manzanilla confirm the cultivar-dependent transcriptional regulation of the olive *GLU* gene during olive fruit development and ripening. Hence, knowledge of the specific *GLU* expression profile for each olive cultivar is critical to determine the optimum harvesting time in order to obtain VOO with the highest phenolic content.

In our study, we have also found that the transcript level of the olive *GLU* gene in olive fruit mesocarp is transcriptionally regulated in response to different abiotic stresses. Low and high temperatures brought about the induction and repression of olive *GLU* gene, respectively. These data are in agreement with those described for the Arabidopsis β-glucosidase gene *AtBG1*, since its expression levels increase when leaves are subjected to cold stress (Lee et al., 2006). The decrease of the expression levels of olive *GLU* gene observed at 35°C is also consistent with the lower content of secoiridoid derivatives in oils extracted from olive fruit pre-treated for 24 h at 30–50°C (García et al., 2001). Darkness treatment of olive fruit from both cultivars produces a decrease in the expression levels of olive *GLU* gene, indicating that light may be implicated in the regulation of its transcription. On the other hand, although plant β-glucosidases have been involved in the defense against herbivores and pathogen attacks (Minic, 2008), this primary response acts at enzyme activity level, being regulated by compartmentalization, since enzyme and natural substrates are differentially located at subcellular level and only meet when cell integrity is disrupted (Morant et al., 2008). Therefore, it is not surprising that olive *GLU* gene is not induced after wounding, which indicates that the regulation at transcriptional level is not operating. In contrast to our data, the transcript increase of olive *GLU* gene in olive fruit mesocarp after olive fruit fly attack has been reported (Corrado et al., 2012), although only one olive fruit developmental stage was used in that study. Finally, higher expression levels of the olive *GLU* gene were detected under water deficit conditions in Picual and Arbequina cultivars, mainly at turning stage. Similar results have been recently reported for the *OeGLU12-like2* gene from cultivar Frantoio (Cirilli et al., 2017). The increase in the olive *GLU* transcript level detected under water deficit conditions, with the corresponding increase in the enzyme activity, could also significantly contribute to explain the higher content of secoiridoid derivatives reported in VOO obtained from olive fruit under water stress conditions (Artajo et al., 2006; Stefanoudaki et al., 2009).

## Conclusion

We have purified the olive recombinant β-glucosidase enzyme (OepGLU). Immunological detection, molecular mass determination and kinetic properties of the recombinant OepGLU strongly indicates that it corresponds to the native olive β-glucosidase enzyme previously purified from olive fruit mesocarp (Romero-Segura et al., 2009), which has been shown as the main enzyme involved in the transformation during VOO processing, of oleuropein and other phenolic glycosides from olive fruit onto their corresponding secoiridoid derivatives present in VOO. However, the contribution of other olive fruit β-glucosidase isoenzymes to oleuropein hydrolysis cannot be discarded. Our results have also shown that olive *GLU* gene expression is not only spatially and temporally regulated in olive fruit, but also is cultivar-dependent and regulated by temperature, light and water regime. This study represents a significant step to elucidate the factors responsible for the phenolic content and profile of VOO. In addition, this information will help in the design of molecular markers for the marker-assisted selection of novel olive cultivars with improved phenolic content and composition in their oils.

## Accession Numbers

The nucleotide sequence reported in this paper for *OepGLU* gene has been submitted to the GenBank/EMBL/DDBJ database with the accession number KX278417.

## Author Contributions

AP and JM-R conceived and designed the study, DV-P and CR-S performed the cloning and qRT-PCR experiments, DV-P and RG-R carried out the immunological studies, DV-P performed the transient expression experiments and the analytical and biochemical studies, MH and JM-R supervised the cloning, qRT-PCR and immunological studies, FV and IG supervised the transient expression experiments, AP supervised the analytical and biochemical studies, JM-R wrote the manuscript. All authors discussed and commented the manuscript.

## Conflict of Interest Statement

The authors declare that the research was conducted in the absence of any commercial or financial relationships that could be construed as a potential conflict of interest.

## Footnotes

1 http://primer3.ut.ee/
