# Supplementary material for: An Oleuropein β-Glucosidase from Olive Fruit Is Involved in Determining the Phenolic Composition of Virgin Olive Oil
Source: Front Plant Sci. 2017 Nov 7;8:1902. doi: 10.3389/fpls.2017.01902 (PMC5682033; doi:10.3389/fpls.2017.01902)
Supplement: Supplementary file 1 [file Data_Sheet_1.pdf]

Supplementary Material

An Oleuropein β-Glucosidase from Olive Fruit Is Involved in Determining the Phenolic Composition of Virgin Olive Oil

David Velázquez-Palmero[1,2], Carmen Romero-Segura[1], Rosa García-Rodríguez[1], M. Luisa Hernández[1], Fabián E. Vaistij[2], Ian A. Graham[2], Ana G. Pérez[1], and José M. Martínez-Rivas[1,*]

[1]Department of Biochemistry and Molecular Biology of Plant Products, Instituto de la Grasa (CSIC), 41013-Sevilla, Spain

[2]Centre for Novel Agricultural Products, Department of Biology, University of York, York YO10 5DD, United Kingdom

***Correspondence:** J.M. Martínez-Rivas : mrivas@cica.es

TABLE S1. Gene accession numbers and sequences of primers pairs used for gene expression analysis by qRT-PCR in the present study.

Gene	Accession number	Sequence	Amplicon size (bp)
OepGLU	KX278417	Forward: 5’-CTAGCGATGGACCAAGGTG-3’<br>Reverse: 5’-AATGGAACTGCTGTAGGCTTG-3’	197
OeUBQ2	AF429430	Forward: 5’-AATGAAGTCTGTCTCTCCTTTGG-3’<br>Reverse: 5’-AAGGGAAATCCCATCAACG-3’	132

Figure S1

```
OepGLU : MDIQSN-VLTITSGSSPTDTSSNGQAAKSTKERIKRSDFPSDFVFGAATASYQVEGAWNE :  59
PtGLU  : MAITSIAHLRVVNANMSIPLARLRVVNANISIPLKRTSFPKKFLFGAGSASYQYEGAAHI :  60
RsGLU1 : ---------------MATQSS--AVIDSNDATRISRSDFPADFIMGTGSSAYQIEGGARD :  43
PiGLU  : ---------------MSSVLP--TPVLPTPGRNINRGHFPDDFIFGAGTSSYQIEGAARE :  43

OepGLU : GGKGMSNWDYFTQSQPGGISDFSNGTIAIDHFNMFKDDVVVMKKLGLKAYRFSLSWPRIL : 119
PtGLU  : DGRGLSVWDVFTKEHPEKIADQSNGDVAQDFYHRYKEDIKSMKEMGLESFRFSISWSRIL : 120
RsGLU1 : GGRGPSIWDTFTHRRPDMIRGGTNGDVAVDSYHLYKEDVNILKNLGLDAYRFSISWSRVL : 103
PiGLU  : GGRGPSIWDTFTHTHPELIQDGSNGDTAINSYNLYKEDIKIVKLMGLDAYRFSISWPRIL : 103

OepGLU : PGGRLCHGVSKEGVQFYNDLIDALLAADIEPYITIFHWDIPQCLQLEYGGFLHERVVQDF : 179
PtGLU  : PNGKISGGINKLGIKFYNNLIDELLANGIKPLVTIYHWDLPQALQDEYGGFLSPKIVDDF : 180
RsGLU1 : PGGRLSGGVNKEGINYYNNLIDGLLANGIKPFVTLFHWDVPQALEDEYGGFLSPRIVDDF : 163
PiGLU  : PGGSINAGINQEGIKYYNNLIDELLANDIVPYVTLFHWDVPQALQDQYDGFLSDKIVDDF : 163

OepGLU : IEYSEICFWEFGDRVKYWITLNEPWSFT-VQGYVAGAFPPNRGVTPKDTEETKKHARLHR : 238
PtGLU  : LEYANLVFKEFGDRVKHWATLNEPNIMT-QQGYVFGAHAPGRCSHFEWN----------- : 228
RsGLU1 : CEYAELCFWEFGDRVKHWMTLNEPWTFS-VHGYATGLYAPGRGRTSPEHVNHPTVQHRCS : 222
PiGLU  : RDFAELCFWEFGDRVKNWITINEPESYSNFFGVAYDTPPKAHALKASRLLVPTTVARPSK : 223

OepGLU : GGGKLLTAFKYGNPGT----EPYKVAHNLILCHAHAVDIYRTKYQESQGGKIGITNCISW : 294
PtGLU  : --------CPAGNSGT----EPYIVGHHLLLCHAAAFQLYKQKYKDDQKGIIGITTATQM : 276
RsGLU1 : TV-APQCICSTGNPGT----EPYWVTHHLLLAHAAAVELYKNKFQRGQEGQIGISHATQW : 277
PiGLU  : PV-R--VFASTADPGTTTADQVYKVGHNLLLAHAAAIQVYRDKFQNTQEGTFGMALVTQW : 280

OepGLU : NEPLTDS-QEDKDAATRGNDFMLGWFVEPVVTGEYPESMIKNVGDRLPKFSEKEEKLVKG : 353
PtGLU  : AIPLNDN-VANLLAASRAIDFNIGWFLHPVVYGEYPQTMRERLGSRLPKFTEKESEMLKQ : 335
RsGLU1 : MEPWDENSASDVEAAARALDFMLGWFMEPITSGDYPKSMKKFVGSRLPKFSPEQSKMLKG : 337
PiGLU  : MKPLNENNPADVEAASRAFDFKFGWFMQPLITGEYPKSMRQLLGPRLREFTPDQKKLLIG : 340

OepGLU : SYDFLGINYYTSTYTS---DDPTKPTTDSYLTDSRTKTSHERNKVPIGAQAGSDWLYIVP : 410
PtGLU  : SFDFIGLNYYSTDYAA-ASSFSVDPVNVSYTTDSRATLSAIKDGVPIGDPTFMSWLHIYP : 394
RsGLU1 : SYDFVGLNYYTASYVTNASTNSSGSNNFSYNTDIHVTYETDRNGVPIGPQSGSDWLLIYP : 397
PiGLU  : SYDYVGVNYYTATYVSSAQP-PHDKKKAVFHTDGNFYTTDSKDGVLIGPLAGPAWLNIVP : 399

OepGLU : WGIYRVMVDMKKRYNDPVIYITENGVDEVNDKSKTST-EALKDDIRIHYHQEHLYYLKLA : 469
PtGLU  : EGILTLLRYVKERYNNPFVMITENGMADENKGSLAEDPMALKDNVRIRYHREHLYYVLEA : 454
RsGLU1 : EGIRKILVYTKKTYNVPLIYVTENGVDDVKNTNLTLS-EARKDSMRLKYLQDHIFNVRQA : 456
PiGLU  : EGIYHVLQDIKENYEDPVIYITENGVYEVNDTAKTLS-EARVDTTRLHYLQDHLSKVLEA : 458

OepGLU : MDQGVNLKGYFIWSLFDNFEWAAGFSVRFGVMYVDYANGRYTRLPKRSAVWWRNFLTKPT : 529
PtGLU  : IKEGVNVGGYYAWTWMDDFEWGSGYTPRFGLNFVDFDN-DLKRTPKDSYFWFKDFLAN-- : 511
RsGLU1 : MNDGVNVKGYFAWSLLDNFEWGEGYGVRFGIIHIDYND-NFARYPKDSAVWLMNSFHKNI : 515
PiGLU  : RHQGVRVQGYLVWSLMDNWELRAGYTSRFGLIHIDYYN-NFARYPKDSAIWFRNAFHKRL : 517

OepGLU : AVPLKNEPEKSEDRRKRLRGST---- : 551
PtGLU  : -------------------------- :   -
RsGLU1 : SKLPAVKRSIREDDEEQVSSKRLRK- : 540
PiGLU  : RIHVNKARPQEDDGAFDTPRKRLRKY : 543
```

FIGURE S1. Comparison of deduced amino acid sequences of *Olea europaea*, *Polygonum tinctorium*, *Rauvolfia serpentina* and *Psychotria ipecacuanha GLU* genes. The sequences were aligned using the ClustalX program and displayed with GeneDoc. Identical and similar residues are shown on a background of black or gray, respectively. Conserved sequence motifs among the family 1 glycosyl hydrolases are framed, which include the two glutamic acid residues involved in the catalytic mechanism, indicated by asterisks. The conserved residues involved in the glucose binding are denoted by triangles. The putative N-glycosylation site of OepGLU is denoted by an arrow. The sequences of a putative signal peptide and a putative nuclear localization signal are underlined with dashed or continuous lines, respectively. The cDNA sequence corresponding to *OepGLU* has been deposited in the GenBank/EMBL/DDBJ database with the accession number KX278417. Accession numbers of sequences included in the alignment are as follows: *P. tinctorium* (PtGLU, AB003089), *R. serpentina* (RsGLU1, AF149311) and *P. ipecacuanha* (PiGLU, AB455576).

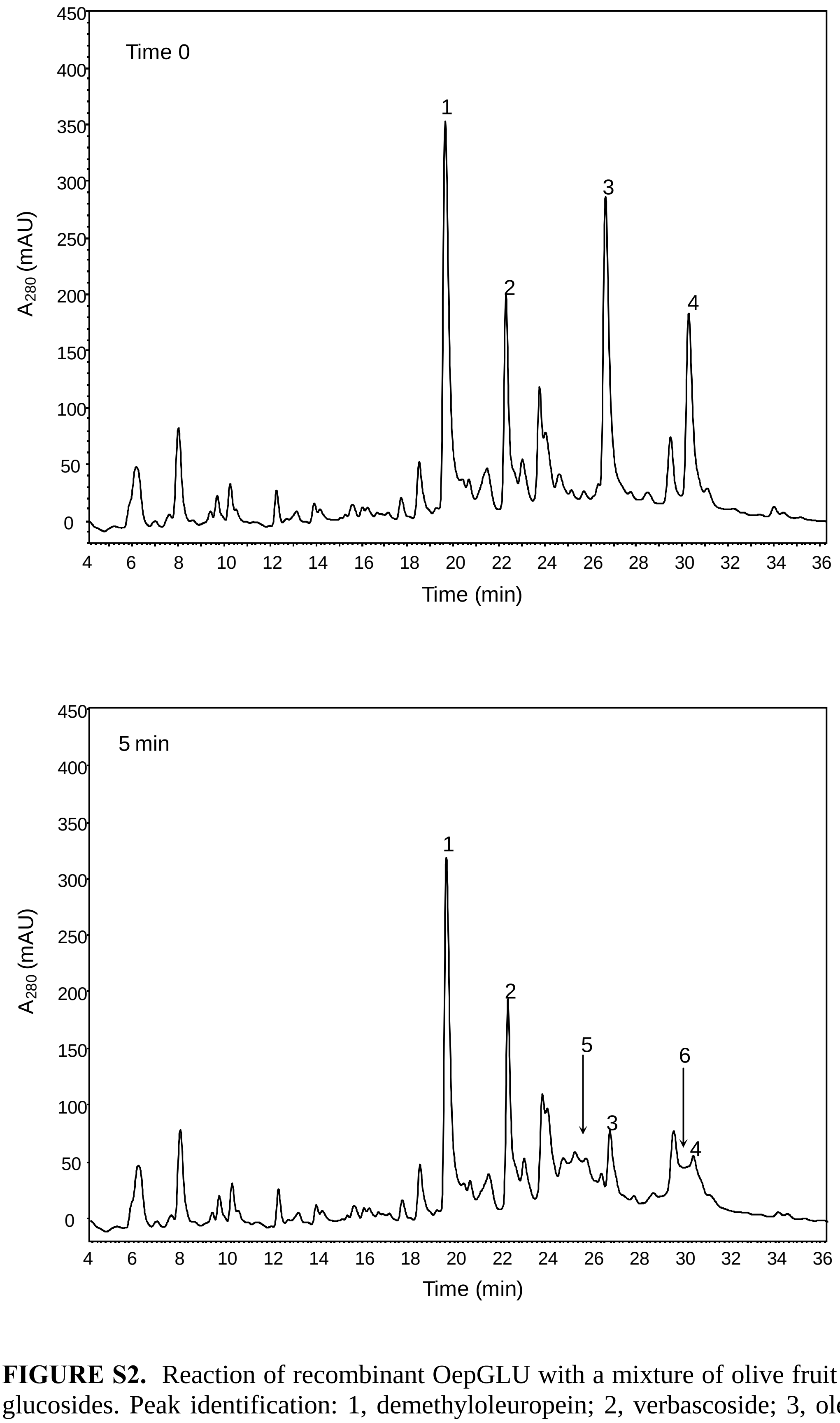

FIGURE S2. Reaction of recombinant OepGLU with a mixture of olive fruit phenolic glucosides. Peak identification: 1, demethyloleuropein; 2, verbascoside; 3, oleuropein; 4, ligstroside; 5, OA-isomers; 6, LA-isomers.

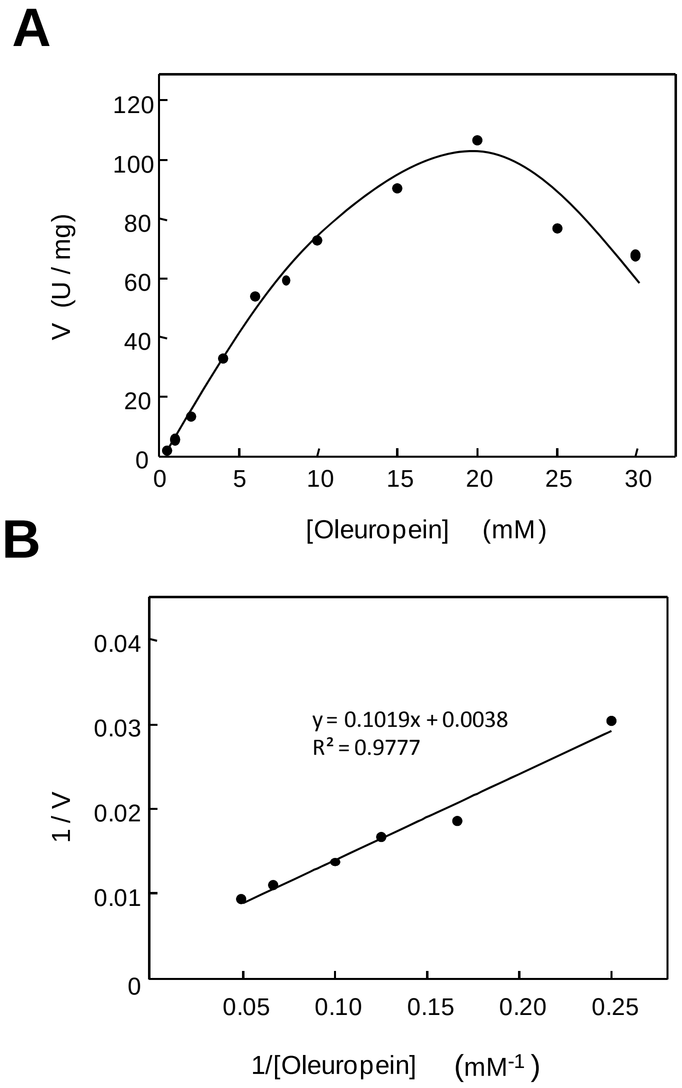

FIGURE S3. Effect of oleuropein concentration on recombinant OepGLU enzymatic activity (A). Lineweaver-Burk plot (B).

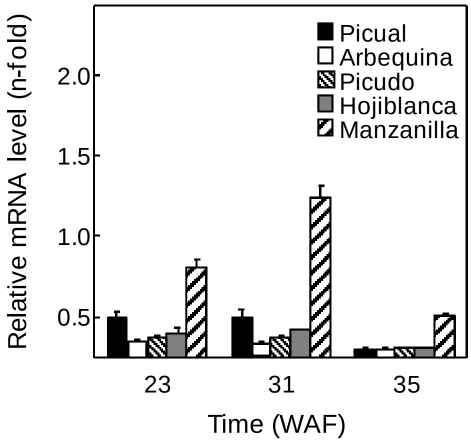

FIGURE S4. Relative expression levels of olive *GLU* gene in olive fruit mesocarp from different cultivars at three ripening stages.
